# Effects of Vaccination Against Coccidiosis on Gut Microbiota and Immunity in Broiler Fed Bacitracin and Berry Pomace

**DOI:** 10.3389/fimmu.2021.621803

**Published:** 2021-06-04

**Authors:** Quail Das, Julie Shay, Martin Gauthier, Xianhua Yin, Teri-Lyn Hasted, Kelly Ross, Carl Julien, Hassina Yacini, Yan Martel Kennes, Keith Warriner, Massimo F. Marcone, Moussa S. Diarra

**Affiliations:** ^1^Department of Food Science, University of Guelph, Guelph, ON, Canada; ^2^Guelph Research and Development Centre, Agriculture and Agri-Food Canada (AAFC), Guelph, ON, Canada; ^3^Ottawa Laboratory (Carling) - Research and Development, Canadian Food Inspection Agency, Ottawa, ON, Canada; ^4^Biological Informatics Centre of Excellence, AAFC, Saint-Hyacinthe, QC, Canada; ^5^Summerland Research and Development Centre, AAFC, Summerland, BC, Canada; ^6^Centre de recherche en sciences animales de Deschambault, Deschambault, QC, Canada

**Keywords:** broilers, bacitracin, berry pomaces, coccidiosis vaccination, blood immunoglobulins, cecal microbiota

## Abstract

Feeding practices have been found to influence gut microbiota which play a major role in immunity of poultry. In the present study, changes in cecal microbiota and humoral responses resulting in the 55 ppm bacitracin (BACI), 1% each of cranberry (CP1) and wild blueberry (BP1) pomace alone or in combination (CP+BP) feeding in broiler Cobb 500 vaccinated or not against coccidiosis were investigated. In the non-vaccinated group, no significant treatment effects were observed on performance parameters. Vaccination significantly affected bird’s performance parameters particularly during the growing phase from 10 to 20 days of age. In general, the prevalence of coccidiosis and necrotic enteritis (NE) was reduced by vaccination (*P* < 0.05). BACI-treated birds showed low intestinal lesion scores, and both CP1 and BP1 feed supplementations reduced *Eimeria acervulina* and *Clostridium perfringens* incidences similar to BACI. Vaccination induced change in serum enzymes, minerals, and lipid levels in 21-day old birds while, levels of triglyceride (TRIG) and non-esterified fatty acids (NEFA) were higher (*P* < 0.05) in CP1 treated non–vaccinated group than in the control. The levels of NEFA were lower in BACI- and CP1-fed birds than in the control in non-vaccinated day 28 old birds. The highest levels of all estimated three immunoglobulins (IgY, IgM, and IgA) were found in the vaccinated birds. Metagenomics analysis of the cecal bacterial community in 21-day old birds showed the presence of *Firmicutes* (90%), *Proteobacteria* (5%), *Actinobacteria* (2%), and *Bacteroidetes* (2%). In the vaccinated group, an effect of BACI was noted on *Proteobacteria* (*P* = 0.03). Vaccination and/or dietary treatments influenced the population of *Lactobacillaceae*, *Enterobacteriaceae, Clostridiaceae*, and *Streptococcaceae* which were among the most abundant families. Overall, this study revealed that besides their beneficial effects on performance, alike bacitracin, berry pomaces in poultry feed have profound impacts on the chicken cecal microbiota and blood metabolites that could be influenced by vaccination against coccidiosis.

## Introduction

Consumers’ demand for antibiotic-free and organic poultry products encourages researchers to find alternative feed additives to control infections in birds (1). However, antibiotic-free and organic poultry production systems can increase risks of birds exposure to pathogens of poultry heath concerns such as *Eimeria, Clostridium perfringens*, avian pathogenic *Escherichia coli*, as well as those having impacts on public health and food safety like *Campylobacter* and *Salmonella enterica* serovars (1, 2). Moreover, modern genetic selection towards lean and large breast muscles, and fast growth rates made broilers susceptible to oxidative reactions that can impact their productivity (3).

Avian coccidiosis is caused by several distinct of *Eimeria* species including, *E. acervulina, E. maxima*, and *E. tenella*, affecting different sites from the duodenum to ceca (4). *Eimeria* infections cause decreased nutrient absorption and egg production as well as a retarded growth rate, a suppression of humoral and cell-mediated immune responses, and a high mortality resulting thus in enormous economic losses (US $3 billion) to the poultry industry worldwide (4). Used litters or chick delivery boxes could be primary sources of *Eimeria* species. The routine prophylactic use of anticoccidials such as ionophores or vaccination with live virulent or attenuated *Eimeria* species have been practiced to control coccidiosis (5). Coccidiosis induces T-cell-associated immune response such as increased levels of IFN-γ. This cytokine has been found to have anticoccidial and adjuvant effects that increase vaccine efficacy in *Eimeria*-infected chicken (6).

Control of *Eimeria* infections also provides concomitant protection against necrotic enteritis (NE) caused by *C. perfringens* (7). This bacterium can produce several different toxins with alpha (α), beta (ß), epsilon (ϵ), and iota (i), and NetB (responsible for the leakage of the intestinal contents) being the major extracellular toxin types (8). The onset of NE is a complex process requiring one or combination of predisposing factors include *Eimeria* co-infection, dietary factors [diets based on wheat, rye, oats or barley; or high levels of animal proteins such as meat meal and fishmeal in feed], indigestible non-starch polysaccharides, stress-causing immunosuppression, and an overall imbalance of commensal microbiota (8). In broilers, *C. perfringens* can lead to significant economic losses of about $6 billion annually (2). This disease has long been controlled by in-feed antibiotics such as virginiamycin and bacitracin. The emergence of drug-resistant oocysts, legislative prohibition on the use of antimicrobial growth promoters (AGP), and non-therapeutic antibiotics used as feed additives, as well as the limited production capacity and costs of live attenuated vaccines, urge the development of alternative coccidiosis and NE control strategies in the poultry industry (9). The composition of gut microbial population is influenced largely by the dietary interventions (10). Gut microbes help in the formation or development of gut structure and morphology, in enhancing immune responses, and offer protection against luminal pathogens, while playing an active role in digestion and utilization of nutrients (10). A balanced gut microbiota protects the mucosal surface of the intestine by preventing the adherence of the pathogens and by producing metabolites. Dietary blueberry was found to change gut microbiota which could help in maintaining immune homeostasis by inhibiting the production of pro-inflammatory cytokines, enhancing the expression of mucin-2 and preventing inflammation (11).

Berry fruit pomaces are a significant source of flavonoids, phenolic acids, and stilbenoids, which exert non-specific effects on living organisms and regulate the activity of enzymes and cell receptors (12). Polyphenol-rich extracts from cranberry fruits displayed potential in increasing energy efficiency, insulin sensitivity, and in decreasing triglyceride (TRIG) and cholesterol (CHO) contents (13). Most importantly, they are powerful antioxidants that improved anti-oxidative responses in the liver (14). The antioxidant components of blueberry, which was positively correlated with the content of polyphenols, increased the short chain fatty acids (SCFA) production (15). Recent chemical analysis indicated that cranberry pomace is a rich source of carbohydrates and fibers (16). These authors suggested that fatty acids, proteins, and essential amino acids in berry pomaces could improve the muscle growth in chicks at the early stages of life. Moreover, the addition of berry pomaces to animal diets positively influenced gut microbiota composition in broiler chickens, by long-term modulations of *Bifidobacterium*, *Rikenellaceae* and *Faecalibacterium*, while decreased in numbers of undesirable ones such as *Synergistaceae*, *Desulfovibrio* and *Fusobacteriaceae* (16).

The development of next generation sequencings and bioinformatics pipelines are allowing to get deep insights into microbiota dynamics with increased coverage and accuracy compared to the traditional non-Sanger first-generation sequencing method. Understanding the mechanisms by which feeding practices and coccidiosis vaccination interact to shape bird’s gut health and immunity is critical to develop a cost-effective alternative feed additive for the antibiotic-free and/or organic broilers production. In the present study, the primary objective was to evaluate effects of feeding bacitracin, cranberry and blueberry pomaces in the presence or absence of a coccidiosis vaccination in broiler. The growth performance parameters, gut health, immune organs weight, immunoglobulin levels, and serum blood metabolites were estimated. Deep metagenomic sequencing was used to investigate the differences in the taxonomic composition of the ir cecal microbiota.

## Materials and Methods

### Pomace Preparation

The preparation and composition in term of phenolic, protein, lipid and carbohydrate of the pomaces used in the present study have been described previously (16, 17).

### Study Design, Broiler Chickens and Treatments

The chicken trial was performed at the Center de Recherche en Sciences Animales de Deschambault, CRSAD, Deschambault, QC, Canada). All experimental procedures performed in this study were approved (protocol #16-AV-314) by the Animal Care Committee of CRSAD, according to guidelines described by the Canadian Council on Animal Care (18). A total of 2700 1-day-old male Cobb 500 broiler birds were randomly allocated to 60 floor pens (45 birds/pen) divided in two coccidiosis vaccination groups (vaccinated and non-vaccinated: 30 pens/group). Each group was randomly allocated to five dietary treatments (6 pens/treatment; 45 birds/treatment) in a complete randomized design. The dietary treatments were (1): basal diet as the control (2); basal diet with 55 ppm bacitracin (BACI) (3); cranberry pomace 1% (CP1) (4); blueberry pomace 1% (BP1); and (5) cranberry 1% and blueberry 1% pomaces combined (CP+BP) administrated from 0 to 30 days of age. Broilers were fed a starter diet from the age of 0 to 10 days, grower diet from 11 to 20 days of age, and a finisher diet from 21 to 30 days of age (19, 20). The starter, grower, and finisher diets were formulated with wheat and corn as the principal cereals; and enriched with soybean, fish, and meat meals as protein sources to meet the nutrient requirements for Cobb 500 (21) (**Table 1**). Birds management, necropsy, data and sample collections for performance parameters, microbiota composition and diversity, blood serum metabolites and immunoglobulin levels were performed as previously described by (19).

**Table 1:** Composition of the diets used in this study.

Ingredient (% of inclusion in diet)	Starter (D0-10)	Grower (D10-20)	Finisher (D20-30)
Wheat ground	30.02	34.941	34.92
Corn	25.2	26.3	30.1
Soybean meal	21	18.8	25
Soybean cake granule	15	12.2	3.2
Flour meat rum Alberta sg	4.8	4.2	3.7
End of corn gluten 60%	1	1	–
Animal fat applicator	1.2	0.8	1.2
Limestone	0.47	0.58	0.62
Liquid methionine	0.33	0.304	0.292
Salt	0.23	0.22	0.22
Baking soda	0.31	0.21	0.21
Px-cr end chicken	0.2	0.2	0.203
Lysine sulphate 70%	0.07	0.088	0.2
Choline liquid 75%	0.06	0.061	0.054
HyD premix (vitamin d3)	0.03	0.036	0.036
Vitamin E 100 000ui	0.05	0.03	0.02
Threonine 98%	0.03	0.024	0.017
Axtra phy 5000l 300f (0.12% p) po	0.01	0.006	0.006
**Calculated Nutrients**			
Crude protein	25.32	23.29	21.30
Metabolisable energy (kcal/kg)	3020.77	3066.11	3153.23
Available phosphorous	0.52	0.49	0.46
Total chloride	0.21	0.21	0.20
Total sodium	0.22	0.19	0.19
Added choline (mg/kg)	396.99	396.99	351.43
Added VitA (UI/kg)	11000.00	10100.00	10100.00
Added VitD (UI/kg)	4988.24	4984.32	4984.32
Added VitE (UI/kg)	80.00	60.00	50.00
Arginine	1.67	1.50	1.38
Lysine	1.38	1.23	1.19
Methi & cys	1.08	1.01	0.94
Methionine	0.67	0.62	0.58
Threonine	0.95	0.87	0.78
Tryptophan	0.33	0.30	0.27
Arg dig v vol	1.53	1.38	1.26
Lys dig v vol	1.18	1.06	1.03
M&C DIG V VOL	0.97	0.90	0.84
Met DIG V VOL	0.64	0.59	0.55
THR DIG V VOL	0.81	0.73	0.66
TRY DIG V VOL	0.28	0.26	0.24
VALDVV/LDV	0.86	0.87	0.82
Total calcium phytase	1	0.96	0.9

### Oocyst Counts of Excreta Samples

Droppings (3 cecal and 3 intestines) per pen (total 60 pens) were collected from different random places on the floor at 13, 16, 20, and 23 day of age using sterile spatulas and kept in separate airtight plastic bags. Samples were immediately stored at 4°C until assayed for total oocyst counts. Homogenized samples (60 pooled cecal and intestinal) were 10-fold diluted with tap water and further diluted with a saturated sodium chloride solution at a ratio of 1:10, counted using McMaster chambers [McMaster chambers, Chalex Corp., Wallowa, OR], and expressed as log-total numbers of oocysts counted per gram of excreta (log10/g) (22).

### General and Gut Health

At each of the days 21 and 28, two birds/pen having average pen weight (12/treatment and 60 per vaccinated or non-vaccinated group for a total of 120 birds) were sacrificed for the necropsy by a veterinary (Services Vétérinaires Ambulatoires Triple-V Inc.) blinded to the treatments. The intestines of all sacrificed birds were examined for evidence of coccidiosis caused by three *Eimeria* spp. (*E. acervulina, E, maxima* and *E. tenella*) and NE (20). Intestines were longitudinally opened to score mucosa on a scale of 0 to 3 for NE lesions for each of the upper gut and lower gut (including ceca). Coccidiosis lesions were scored on a scale of 0 to 4 for each of *E. maxima* which induces bleeding in the middle of the small intestines, mucosa; *E. tenella* causing severe inflammation of ceca, and *E. acervulina* causing white plaques in the duodenum. The body weights of killed birds were determined. Birds were inspected at least twice a day, and mortalities or culls were removed and necropsied. The mortality rate was calculated based on the average mortality in each pen from day 0 to 30.

### Collection of Immune Organs

On day 21, four organs (liver, spleen, bursa of Fabricius, and thymus) from the 120 sacrificed birds (two birds/pen, twelve birds/treatment having average pen weight) were surgically removed and weighted. Ratios of organ weight/bodyweight were estimated.

### Blood Antibodies and Metabolites Measurement

Blood samples were collected *via* wing vein from sacrificed birds at days 21 and 28, and kept at room temperature for 2 hours followed by centrifugation for 10 min at 580 X g. Serum of two birds from each pen (repetition) at each time point (chicken age) were pooled together (12 birds, 6 repetitions/treatment). Serum immunoglobulins (Igs) IgY/IgG, IgM, and IgA levels were measured on flat-bottom 96-well plates by enzyme-linked immunosorbent assay (ELISA) as previously described (23). To determine dietary treatments and vaccination effects, 19 blood serum parameters were estimated at the Animal Health Laboratory (University of Guelph, Guelph, ON, Canada) as previously described (20).

### DNA Extraction and Metagenomic Sequencing

DNAs were extracted from cecal samples of 80 birds from 40 pens (two birds/pen, eight birds/treatment having average pen weight). Each cecal samples were aseptically collected, transferred to sterile Whirl-Pak plastic bags (Nasco, Fort Atkinson, WI), immediately frozen (-20°C), and transported to the laboratory for taxonomy analysis. DNA isolation from the cecal samples was performed using QIAamp DNA Stool Mini Kit (Qiagen, Venlo, Netherlands) with modification as previously described (24).

### Metagenomic Sequencing and Data Processing

Metagenomic sequencing and its quality control were performed at the Genome Quebec Innovation Centre (Montréal, QC, Canada). TruSeq DNA libraries were prepared and samples were run on an Illumina HiSeq2000 platform, to generate 2 × 100 base paired-end (PE) sequences (25). FastQC version 0.11.8 and MultiQC version 1.6 were used to generate summary reports about the sequence quality of each sample and the data set as a whole. Trimmomatic version 0.38 was used with recommended settings to remove adapters, remove leading and trailing bases below a Phred score of 3, using a sliding window to cut reads when the average base quality drops below a Phred score of 15, and dropping reads less than 36 bases long after trimming.

### Taxonomic Profiling of Reads

For microbiota analysis, reads passing quality filters from Trimmomatic were classified using Kraken 1.0 and the default Kraken database. The kraken-filter script was used with a threshold score of 0.05 to enhance the accuracy of taxonomic assignments. Reports were generated with the kraken-report script.

### Alpha/Beta Diversity of Kraken Taxonomic Plots

The calculation of the cecal samples’ alpha diversity was performed using the Kraken analysis data from which microorganisms count at the domain level and bacteria count at both the phylum and family levels were extracted. The Shannon diversity index was computed to determine both the richness and evenness of the samples using Kraken’s taxonomic assignments as operational taxonomic units (OTU). The association between microbial diversity and treatment groups was tested by multivariate statistics, and ANOSIM from the R package vegan v2.4–1. Sample variation between groups or beta-diversity was performed with Bray-Curtis dissimilarity as implemented in the R package vegan v2.4-1. For all the statistical analyses performed, a *P-*value < 0.05 was considered significant.

### Statistical Analysis

The experiment was performed using a complete randomized design: 2 vaccine groups (vaccinated or non-vaccinated) each them having 5 feed treatments (Control, BACI, CP1, BP1, and CP+BP). Effects of vaccination and dietary treatments on growth performance, oocyst counts (Log10/g), blood parameters and the relative microbial abundances and diversity were analyzed as a complete randomized design using the General Linear Mixed Model (GLMM) procedure of SAS 9.4 (26). As prevalence data are ordinal variables, the Cochran-Mantel-Haenszel test of association and logistic analysis (proportional odds model) were used to determine the relationship between vaccination or diet and gut lesions using the FREQ procedures of SAS. Diets (treatments) and vaccination (yes or no) were used as sources of variation and the individual pens as experimental units. The Least significant difference test was used to separate means whenever the *F*-value was significant. A *P* value of 0.05 was used to declare significance.

## Results

### Composition of the Studied Pomaces

The phenolic, carbohydrate, lipid, and mineral contents of used cranberry and blueberry pomaces were described previously by Ross et al. (17) and a detailed composition of the cranberry pomace has been recently reported (16). The cranberry pomace contained 46.3% and 15.5% coarse and fine fibers, respectively while the low-bush blueberry pomace contained 22.0% and 27.9% coarse and fine fibers. The most abundant amino acids (w/w) in these pomaces were glutamic acid (> 0.80%) and aspartic acid (> 0.50%); arginine and leucine were present each at about 0.40% (w/w). Essential amino acids including lysine, threonine, and methionine that influence growth of bird’s muscle at an early age were present at about 0.31, 0.18, and 0.1%, respectively in both pomaces. The pomaces were rich in unsaturated fatty acids. The most abundant fatty acid was palmitic acid 16:0; lowbush blueberry pomace contained 16.7% more palmitic acid than cranberry pomace (9.8%). Conversely, oleic acid 9c-18:1, linoleic acid 18:2n-6, and linolenic acid 18:3n-3 levels were all higher in cranberry pomace (12.5%, 39.4%, and 23.3%, respectively) than in blueberry pomace (6.0%, 34.5%, and 16.4%).

### Growth Performances

Interactions between the coccidiosis vaccine and dietary treatments (*P* < 0.01) were found on body weight (BW) during the growing period (10-20 days of age). In general, the BW was lower (*P* < 0.001) in the vaccinated group than non-vaccinated chickens. In the non-vaccinated group, similar BW was observed with the BACI, CP1, and CP+BP treated birds along with the control, while in the vaccinated group only BACI-feed supplementation showed a high (*P* = 0.001) BW compared to control in the growing period. As expected, similar effects of vaccination, and treatment were also noted on average daily gain (ADG) during this growing phase. BACI treatment improved (*P* < 0.05) the ADG in the vaccinated group during both the starting (0-10 days) and the growing (10-20 days) periods. However, a significant increase for the average daily feed intake (ADFI) (*P* < 0.05) was observed only in the vaccinated group during the finishing period (20-30 days). In this study, vaccination against coccidiosis increased the feed efficiency (FE) during the growing periods (*P* < 0.05), with a significant effect being observed in BACI-treated birds only. Overall, BACI treatment was found to improve (reduce) the FE (1.49) in the non-vaccinated group (*P* < 0.05) compared to all the treatments. No effect of vaccination was observed on mortality however, higher mortalities were observed in CP+BP and BP1 treatments in the vaccinated group, and in CP1 in the non-vaccinated group (*P* < 0.05, **Table 2**). BACI-fed group resulted in the lowest mortality in both non-vaccinated (2.22) and vaccinated (2.59) treated birds compared to all other treatments (*P* < 0.05, **Table 2**). The combination of CP+BP resulted in a low BW, and a high FE and mortality rate during the growing phase in the vaccinated group compared to non-vaccinated broilers.

**Table 2:** Effects of berry products and bacitracin on coccidiosis vaccinated or non-vaccinated broiler growth performances^1^.

Parameters	No					Yes					SEM	*P* value		
Vaccination	Control	BACI	CP1	BP1	CP+BP	Control	BACI	CP1	BP1	CP+BP		Vac	Treat	V X T
**Bodyweight, kg/bird**														
Day 0**^A^**	0.04	0.04	0.04	0.04	0.04	0.04	0.04	0.04	0.04	0.04	0.00	0.0445	0.9807	0.6343
Day 10	0.30	0.31	0.30	0.30	0.30	0.30	0.31	0.37	0.30	0.29	0.02	0.3884	0.4578	0.4212
Day 20**^ABC^**	0.98	0.98	0.98	0.96	0.98	0.91^B^	0.97^A^	0.89^BC^	0.90^BC^	0.88^C^	0.01	<.0001	0.0015	0.0104
Day 30	2.00	2.06	2.01	1.99	2.01	1.98	2.07	2.00	1.99	1.98	0.03	0.5285	0.0833	0.9602
**Daily Gain, kg/bird**														
Day 0-10**^B^**	0.03	0.03	0.03	0.03	0.03	0.026^B^	0.027^A^	0.025^BC^	0.026^B^	0.025^C^	0.027	0.3426	0.0005	0.275
Day 10-20**^ABC^**	0.07	0.07	0.07	0.07	0.07	0.06^B^	0.066^A^	0.058^BC^	0.059^BC^	0.057^C^	0.066	<.0001	0.0045	0.0052
Day 20-30**^A^**	0.09	0.10	0.10	0.10	0.09	0.10	0.10	0.10	0.10	0.10	0.101	0.0018	0.3919	0.4471
Day 0-30	0.06	0.07	0.07	0.06	0.06	0.06	0.07	0.06	0.06	0.06	0.067	0.586	0.0691	0.9357
**Feed efficiency, FE kg/Kg**														
Day 0-10**^B^**	1.22	1.19	1.23	1.26	1.24	1.23^A^	1.18^B^	1.24^A^	1.24^A^	1.23^A^	1.194	0.8627	0.0147	0.8031
Day 10-20**^AC^**	1.43	1.45	1.43	1.46	1.43	1.57	1.43	1.56	1.57	1.63	1.446	<.0001	0.1033	0.0458
Day 20-30	1.76	1.60	1.77	1.76	1.79	1.72	1.67	1.68	1.72	1.68	1.604	0.108	0.0579	0.2285
Day 0-30**^B^**	1.56	1.49	1.56	1.58	1.58	1.6^A^	1.51^B^	1.58^AB^	1.6^A^	1.6^A^	1.492	0.1032	0.0041	0.9966
**Mortality (%)^B^**	4.07	2.22	7.04	2.96	5.55	4.81^AB^	2.59^B^	4.07^AB^	6.67^A^	7.41^A^	2.22	0.307	0.0121	0.0701

^1^Each value represents the mean of six replicates (n = 6 pens of at least 45 chickens/pen). Control (basal diet), BACI (basal diet supplemented with 55 ppm bacitracin); basal diet supplanted with cranberry pomace 1% (CP1); blueberry pomace 1% (BP1); and cranberry 1% + blueberry 1% pomaces together (CP+BP). Different superscripted capital letters within a row indicate significant differences at P < 0.05. Vac, the main effect of vaccination; Treat, main effects of treatments; V X T, the interaction between vaccination and treatments; ^A^ indicates significant P value due to vaccination, ^B^ indicates significant P value due to treatments, ^C^ indicates significant P value due to interaction of vaccination and treatments.

### Oocyst Count

**Figure 1:**
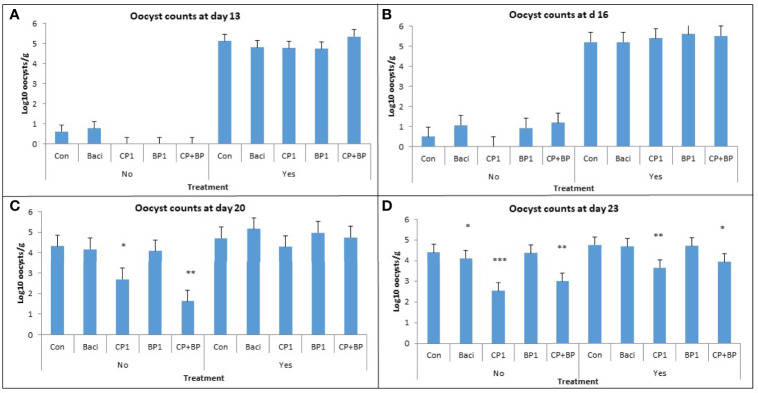
Effects of vaccination and dietary berry pomaces on oocyst counts in intestinal excreta at **(A)** 13, **(B)** 16, **(C)** 20, and **(D)** 23 days old broilers. Birds not vaccinated (No) and vaccinated (Yes) against coccidiosis; Control, birds fed basal diet; BACI, birds fed basal diet with 55 ppm bacitracin; birds fed basal diet with 55 ppm bacitracin; basal diet supplanted with cranberry pomace 1% (CP1); blueberry pomace 1% (BP1); and cranberry 1% + blueberry 1% pomaces together (CP+BP). * indicate statistically significant differences (* means P ≤ 0.05; ** means P ≤ 0.01; *** means P ≤ 0.001).

The effects of vaccination and dietary treatments on *Eimeria* oocyst counts at 13, 16, 20, and 23 days of age are illustrated on **Figure 1**. Vaccinated birds showed a higher oocyst count than the non-vaccinated group at days 13, 16, and 20 (*P* < 0.001). Significant treatment effects of berry pomaces on oocyst counts occurred at days 20 and 23 (*P* < 0.05). In the non-vaccinated group, the lowest count was found in birds receiving CP1 and CP+BP compared to the control at day 20. The lowest oocyst count in both vaccinated and non-vaccinated groups was observed in CP1- and CP+BP-fed birds at day 23 (**Figure 1**). This data suggest that berry pomace may be effective in reducing oocyst counts in the bird’s gut.

### General and Intestinal Health

Gross examination revealed that the general health was good; bones, cartilage, and muscle quality were adequate. No spondylolisthesis, valgus and varus deviations of tibiotarsis, rotated tibia, and runts were observed. Also, no signs of active infections were detected. Intestinal examination showed in general, subclinical (minor low lesion scores) coccidiosis and NE only in day 21-old birds while little or no such intestinal lesions were found at day 28.

Coccidiosis: At day 21, coccidiosis was more prevalent in non-vaccinated than vaccinated birds, with *E. acervulina* followed by *E. tenella* being the predominant causative *Eimeria* species.

**Figure 2:**
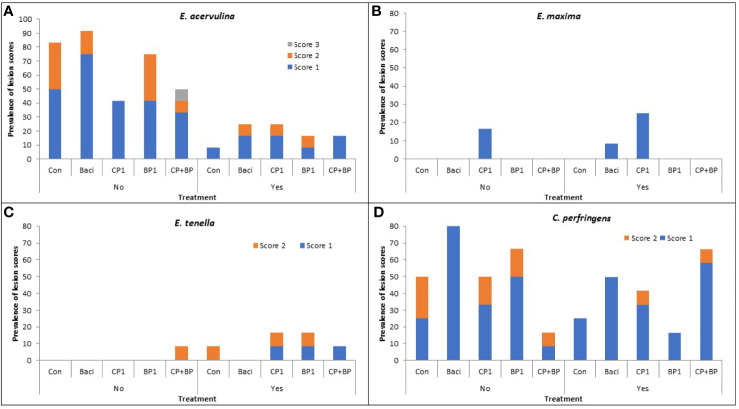
Effects of dietary treatment on the severity and prevalence of coccidiosis lesions due to **(A)** *E. acervulina*, **(B)** *E. maxima*, **(C)** *E. tenella*, and **(D)** necrotic enteritis due to *C. perfringens* in both vaccinated (Yes) or non-vaccinated (No) chickens at 21 day of age. Control, birds fed basal diet; BACI, birds fed basal diet with 55 ppm bacitracin; basal diet supplanted with cranberry pomace 1% (CP1); blueberry pomace 1% (BP1); and cranberry 1% + blueberry 1% pomaces together (CP+BP).

*E. acervulina:* Regardless of treatments, vaccination significantly reduced (*P* < 0.05) the prevalence and severity of intestinal lesions due to *E. acervulina* (**Figure 2A**). In the non-vaccinated group, lesions were noted in 68% of birds. The highest lesion incidence was observed in the BACI-fed birds with a lesion score of 1 (a maximum of 5 lesions per cm^2^ mainly in the duodenum). Lesion scores of 2 (several lesions in the duodenum and/or jejunum, but not coalescent) were observed in the BP1 and control with an incidence of 33%; while only a bird (1.66%) with a lesion score of 3 (numerous coalescent lesions) was observed only in the CP+BP treatment group (**Figure 2A**).

In the vaccinated group, 18% of birds showed intestinal lesions due to *E. acervulina* with an incidence of 8.33% in the control and BP1 group, while 16.67% incidence found in BACI, CP1 and CP+BP treated birds. Lesion score severity of 2 was found only in the BACI, CP1 and BP1 treatments with an incidence of 8.33%.

*E. maxima:* Low incidences of coccidiosis due to *E. maxima* were observed, with 16.67% and 25% being found in the non-vaccinated and vaccinated birds. Among all treatments, CP1-fed birds showed a prevalent of lesion score of 1 (**Figure 2B**).

*E. tenella*: No significant effects of vaccination or treatments were observed for intestinal lesions due to *E. tenella* (**Figure 2C**).

*Necrotic enteritis (NE)*: Vaccination against coccidiosis prevented NE (*P* < 0.05). However, the lowest proportion of birds showing NE was recorded in CP+BP fed birds in the non-vaccinated group while the highest NE incidence was observed with the same feed treatment in vaccinated birds. No lesion scores greater than 2 was observed. In the non-vaccinated group, 100% of birds fed BACI showed the lesion score of 1, however, no score of 2 was observed. Birds in the control group showed the highest incidences (25%) in the non-vaccinated group, with the lowest (8.33%) observed with the (CP+BP) treatment. Both CP1 and BP1 feeding resulted in similar incidences (16.67%) (**Figure 2D**). In the vaccinated group, BP1-fed birds exhibited the lowest NE incidence (6.67% with a score of 1). The highest incidences of NE were observed in CP+BP-fed birds (58.33%), followed by BACI (50%) and CP1 (33.33%). Incidences of birds showing NE scores of 2 were observed only in the CP1- and CP+BP-treated and vaccinated birds.

### Immune Organ Weights

The weights of different organs of 21-day old broiler chickens fed bacitracin and berry pomaces are shown on **Table 3**. Overall, significant effects (*P* < 0.05) of vaccination was observed on the weight of birds and all four organs, while feeding with either bacitracin or different berry pomaces did not significantly affect the weights of the immune organs of broiler chickens. In general, vaccination significantly reduced the organ weights compared to non-vaccinated birds. No significant vaccination or dietary treatment effects were observed on the organ Indexes (organ weight/bodyweight). While treatment with bacitracin and berry pomaces reduced the liver index of broilers due to vaccination, the liver index in the CP+BP combination group tended to increase in vaccinated group compared to non-vaccination.

**Table 3:** The effect of bacitracin and berry pomace extracts on immune organs weights of 21-days old broilers receiving or not a coccidiosis vaccine^1^.

Parameters	Non-vaccinated					Vaccinated					SEM	*P* value		
Weight (g)	Control	Baci	CP1	BP1	CP+BP	Control	Baci	CP1	BP1	CP+BP		Vac	Treat	V XT
**Body weight (BW)**	1169.58	1172.50	1187.08	1123.75	1166.67	1020.83	1034.17	1041.67	1057.08	1024.17	29.01	<.0001	0.93	0.58
Liver	34.93	34.00	35.30	32.68	33.15	27.02	28.76	28.45	30.47	31.94	1.49	<.0001	0.87	0.11
Bursa	15.75	15.25	15.00	14.50	15.50	14.42	14.25	15.00	14.75	14.50	0.13	0.02	0.78	0.24
Spleen	1.32	1.17	1.27	1.09	1.23	0.93	1.15	0.99	1.12	1.02	0.09	0.00	0.99	0.11
Thymus	1.32	1.17	1.27	1.09	1.23	0.93	1.15	0.99	1.12	1.02	0.41	0.03	0.34	0.79
**Index: Organ/BW**														
Liver	2.97	2.91	2.97	2.90	2.84	2.64	2.77	2.77	2.91	3.13	0.12	0.27	0.60	0.07
Bursal	0.20	0.18	0.18	0.16	0.17	0.19	0.18	0.19	0.17	0.19	0.01	0.38	0.16	0.88
Spleen	0.11	0.10	0.11	0.10	0.11	0.09	0.11	0.10	0.11	0.10	0.01	0.51	0.98	0.18
Thymus	0.57	0.60	0.58	0.60	0.58	0.57	0.68	0.59	0.59	0.57	0.03	0.45	0.20	0.67

^1^Each value represents the least square mean (LSmean) of six replicates (n = 6 pens of at least 45 chickens/pen). Control (basal diet), BACI (basal diet supplemented with 55 ppm bacitracin); basal diet supplanted with cranberry pomace 1% (CP1); blueberry pomace 1% (BP1); and cranberry 1% and blueberry 1% pomaces together (CP+BP). Vac, the main effect of vaccination; Treat, main effects of treatments; V X T, the interaction between vaccination and treatments.

### Blood Metabolites

Of the 19 blood serum metabolites screened, significant interactions between dietary treatments and vaccination were observed for minerals [(magnesium (Mg), and phosphorous (P)], glucose (GLU), and NEFA levels at day 21. Vaccination effects were observed on levels of several metabolites including enzymes such as alanine aminotransferase (ALT), aspartate aminotransferase (AST), and Gamma-glutamyl transferase (GGT) as well as minerals such as calcium (Ca) and P (minerals); all lipids markers, and globulin (GLO). In the non-vaccinated group, lower levels of both ALT and AST were observed in the BP1 and CP+BP-treated birds than in control (*P* < 0.05). In contrast, the lowest levels of GGT and Ca were found in the vaccinated control birds. Feed supplementation with BP1 reduced the GLU level in vaccinated birds (*P* < 0.05). At day 21, vaccination affected the level of all screened lipids (*P* < 0.01), however, dietary treatment effects were observed only on the TRIG and NEFA levels. The lowest levels of both TRIG (*P* = 0.028) and NEFA (*P* = 0.025) were observed in BP1 and CP+BP-treated birds in the vaccinated group compared to control. The concentration of GLO was higher in BACI and all berry pomace treated groups compared to control (*P* < 0.05) (**Table 4**).

**Table 4:** Blood serum metabolites level of broiler chickens showing significant dietary treatment and/or vaccination effects.

Age	Blood metabolites		Vaccination	Treatments					SEM	Effects		
				Control	BACI	CP1	BP1	CP+BP		Vac	Treat	V XT
21	Serum enzymes (U/L)	ALT	No	2.33	2.50	1.83	1.00	1.00	0.61	*	ns	ns
			Yes	2.83	3.00	2.83	2.00	4.00				
		AST	No	200.00	218.33	232.00	196.17	190.67	24.27	*	ns	ns
			Yes	245.00	300.33	231.00	234.17	205.17				
		GGT	No	7.67	8.67	8.67	8.00	8.00	1.52	*	ns	ns
			Yes	3.00	6.67	3.83	10.00	4.00				
	Mineral (mmol/L)	Ca	No	2.46	2.44	2.56	2.46	2.48	0.08	*	ns	ns
			Yes	2.29	2.46	2.37	2.35	2.34				
		Mg	No	0.93	0.90	1.05	0.93	0.93	0.06	ns	ns	*
			Yes	1.03	1.20	0.97	1.00	0.92				
		P	No	2.21	2.05	2.50	2.25	2.24	0.15	*	ns	*
			Yes	2.41	2.98	2.48	2.41	2.35				
	Carbohydrate (mmol/L)	GLU	No	15.15	14.75	15.48	15.52	15.08	0.50	ns	ns	*
			Yes	14.62	16.48	15.62	13.70	15.22				
	Lipid (mmol/L)	CHOL	No	2.79	2.76	3.02	2.80	2.97	0.12	**	ns	ns
			Yes	2.27	2.47	2.35	2.34	2.38				
		HDLC	No	2.16	2.09	2.18	2.12	2.30	0.10	**	ns	ns
			Yes	1.79	1.94	1.79	1.90	1.91				
		TRIG	No	0.72	0.82	1.00*	0.88	0.87	0.08	*	*	ns
			Yes	0.70	0.90	0.88	0.62	0.65				
		NEFA	No	0.63	0.68	1.03*	0.86	0.88	0.08	**	*	*
			Yes	0.61	0.63	0.71	0.50	0.48				
	Protein (g/L)	Glo	No	11.83	11.00	12.17	12.17	11.50	0.55	*	ns	ns
			Yes	11.67	12.67	12.17	12.50	13.33				
28	Serum enzymes (U/L)	ALP	No	7337.50	7731.50	8996.67	8525.17	4894.83	1360.63	*	ns	ns
			Yes	9159.83	7325.83	11156.33	11429.00	8481.00				
	Lipid (mmol/L)	TRIG	No	0.70	0.60	0.57	0.60	0.67	0.04	ns	ns	*
			Yes	0.57	0.57	0.63	0.72	0.63				
		NEFA	No	0.80	0.52	0.56	0.60	0.65	0.05	ns	*	ns
			Yes	0.54	0.54	0.60	0.73	0.63				
	Protein (g/L)	TP	No	26.17	24.00	24.17	25.50	25.00	0.69	*	ns	ns
			Yes	25.83	27.50	25.17	25.50	25.67				
		Glo	No	12.67	12.33	11.33	12.33	12.67	0.54	*	ns	ns
			Yes	13.67	15.00	13.00	12.67	12.33				
		A/G Ratio	No	1.07	0.97	1.14	1.07	0.99	0.06	*	ns	ns
			Yes	0.91	0.84	0.95	1.02	1.08				

^1^Basal diet (Control); a basal diet supplemented with 55 ppm (BACI), cranberry pomace 1% (CP1); blueberry pomace 1% (BP1); and cranberry 1% and blueberry 1% pomaces together (CP+BP). Vac, the main effect of vaccination; Treat, main effects of treatments; V X T, the interaction between vaccination and treatments. Asterisks indicate statistically significant differences level: *P ≤ 0.05, **P ≤ 0.01 and ns, not significant (P > 0.05). Blood metabolites showing significant effect (P < 0.05) were included in this table.

Bird’s age (sampling day) effect was observed on all protein levels. Moreover, the levels of AST, GGT, CHO, and HDLC were higher at day 28 than at day 21 of age (*P* < 0.05). Vaccination affected the level of all proteins (*P* < 0.01). The highest level of TP and GLO, and the lowest level of AG ratio were noted in the vaccinated group, particularly in BACI-treated birds (**Table 4**). Interactions between vaccine and dietary treatments were observed on the level of TRIG (*P* = 0.047). In non-vaccinated birds the concentration of TRIG was 19% lower in the CP1-fed birds, however higher in the vaccinated group than in control birds. The effect of treatment was observed only on the NEFA in non-vac group, the concentrations of NEFA were significantly decreased in BACI and CP1-treated birds (35% and 29%, respectively).

### Quantification of Immunoglobulins (Ig) in Sera

**Figure 3:**
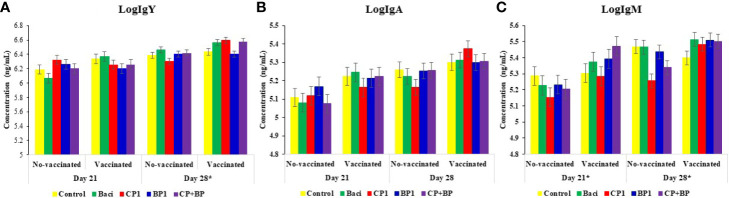
Concentrations (ng/mL) of immunoglobulins **(A)** IgY, **(B)** IgA, and **(C)** IgM in blood sera of broilers at both days 21 and 28 of ages. Birds were treated with basal diet, bacitracin, different berry pomaces administrated *via* feed. Data represent least-square means ± SEM of 6 replicates**/**treatment (n = 6 pens of at least 45 chickens/pen) arranged in a complete randomized design. Asterisks indicate statistically significant differences in vaccination. *indicate statistically significant differences for both vaccination and interaction between vaccination and treatments (vac X treat) at the level of *P* < 0.05.

Effects of BACI, CP1, BP1, and CP+BP in feed s were evaluated at day 21 and 28 on the serum IgY (IgG), IgM, and IgA levels in broiler chickens receiving or not a coccidiosis vaccine (**Figure 3**).

Although, no dietary treatment effect was observed on any Igs, significant effects of vaccination and dietary treatments were found for both IgY and IgM on both day 21 and day 28 (*P* < 0.05). Higher levels of all three Igs were quantified in vaccinated birds than no-vaccinated ones (*P* < 0.05). Moreover, the titers of all three immunoglobulins were higher at day 28 than at day 21 (*P* < 0.001). In general, IgY was the most abundant antibody of all three Igs with the highest titer being observed in CP1 treatment at day 28 (*P* = 0.06).

### Taxonomy

In this study, bioinformatic approach, Kraken pipeline was used to access the taxonomic classifications (bacteria, viruses, and archaea) of the studied broiler cecal microbiota, where it detected 35 phyla with 324 families.

**Figure 4:**
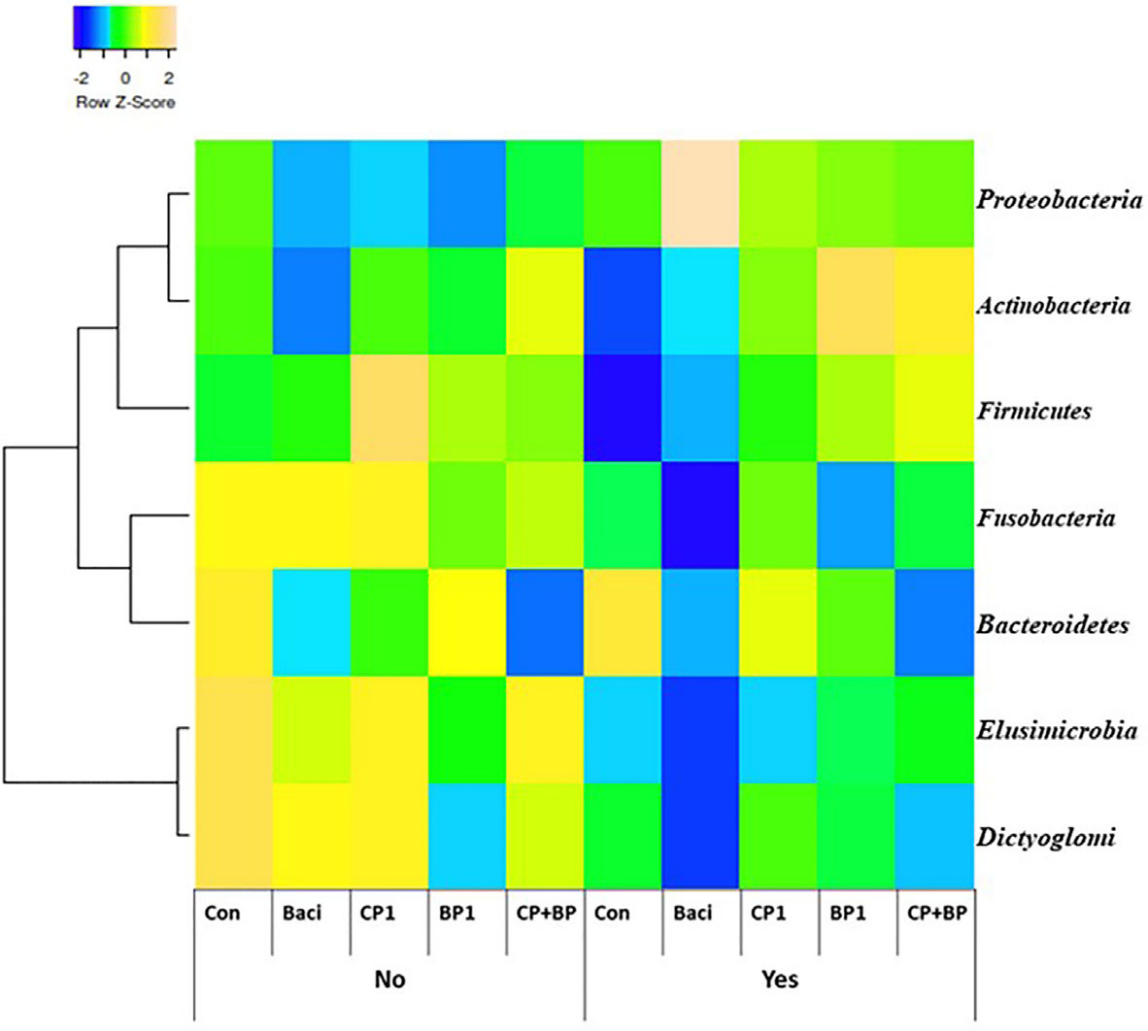
Cecal bacterial taxa at the phylum level in broiler chicken fed bacitracin (55 ppm), 1% of each pomace of cranberry (CP1) and blueberry (BP1) alone or their combination (CP+BP). Birds were vaccinated not vaccinated against coccidiosis.

At the domain level, only viruses showed significant (*P* = 0.024) vaccination effect (**Table 5** and **Figure 4**) but no treatment or interaction between feed and vaccination was observed. *Herpesvirales, Ortervirales, Caudovirales, Bunyavirales* were the predominant virus detected while *Euryarchaeota, Thaumarchaeota*, and *Crenarchaeota* were the most abundant archaea in broiler’s ceca.

Cecal bacterial taxa at the phylum level in broiler chicken fed bacitracin and berry pomaces in birds vaccinated and not vaccinated against coccidiosis are shown on **Figure 4**. In the non-vaccinated group, populations of *Fusobacteria, Elusimicrobia* and *Dictyoglomi* were more abundant in the control birds and those fed BACI and CP1. Population of *Actinobacteria* and *Firmicutes* were predominantly present in BP1 and CP+BP fed birds in the vaccinated group.

**Table 5:** Microbial composition in the ceca digesta of 21-day-old broilers at domain and phylum levels significantly (*P* < 0.05) affected by studied dietary treatments.

	Taxonomy	Vac	Treatments					SEM	*P* values		
			Control	BACI	CP1	BP1	CP+BP		Vac	Treat	V X T
Domain	Virus	No	3.89	3.79	3.86	3.66	3.97	0.16	0.02	0.48	0.31
		Yes	4.36	4.22	3.92	4.05	3.86				
	Bacteria	No	6.73	6.71	6.94	6.81	6.78	0.08	0.51	0.12	0.46
		Yes	6.57	6.77	6.57	6.83	6.84				
	Archea	No	3.06	2.91	3.04	2.86	2.99	0.05	0.30	0.24	0.19
		Yes	3.03	2.99	2.98	3.03	3.00				
Phyla	*Actinobacteria*	No	5.04	4.90	5.04	4.99	5.13	0.11	0.62	0.29	0.57
		Yes	4.87	4.96	5.08	5.22	5.17				
	*Bacteroidetes*	No	4.50	3.63	3.92	4.37	3.35	0.47	0.51	0.12	0.46
		Yes	4.56	3.49	4.32	4.03	3.39				
	*Proteobacteria*	No	5.38	5.08	5.13	5.03	5.20	0.13	0.00	0.30	0.04
		Yes	5.36	5.96	5.48	5.43	5.40				
	*Fusobacteria*	No	2.91	2.91	2.91	2.81	2.86	0.13	0.03	0.84	0.89
		Yes	2.72	2.54	2.82	2.66	2.72				
	*Dictyoglomi*	No	1.06	0.94	0.96	0.48	0.85	0.20	0.01	0.47	0.50
		Yes	0.57	0.26	0.68	0.53	0.44				
	*Elusimicrobia*	No	1.06	0.96	1.01	0.82	1.02	0.16	0.03	0.90	0.90
		Yes	0.74	0.61	0.74	0.77	0.81				
	*Firmicutes*	No	6.63	6.66	6.90	6.75	6.72	0.10	0.14	0.07	0.53
		Yes	6.40	6.54	6.67	6.75	6.79				

**Figure 5:**
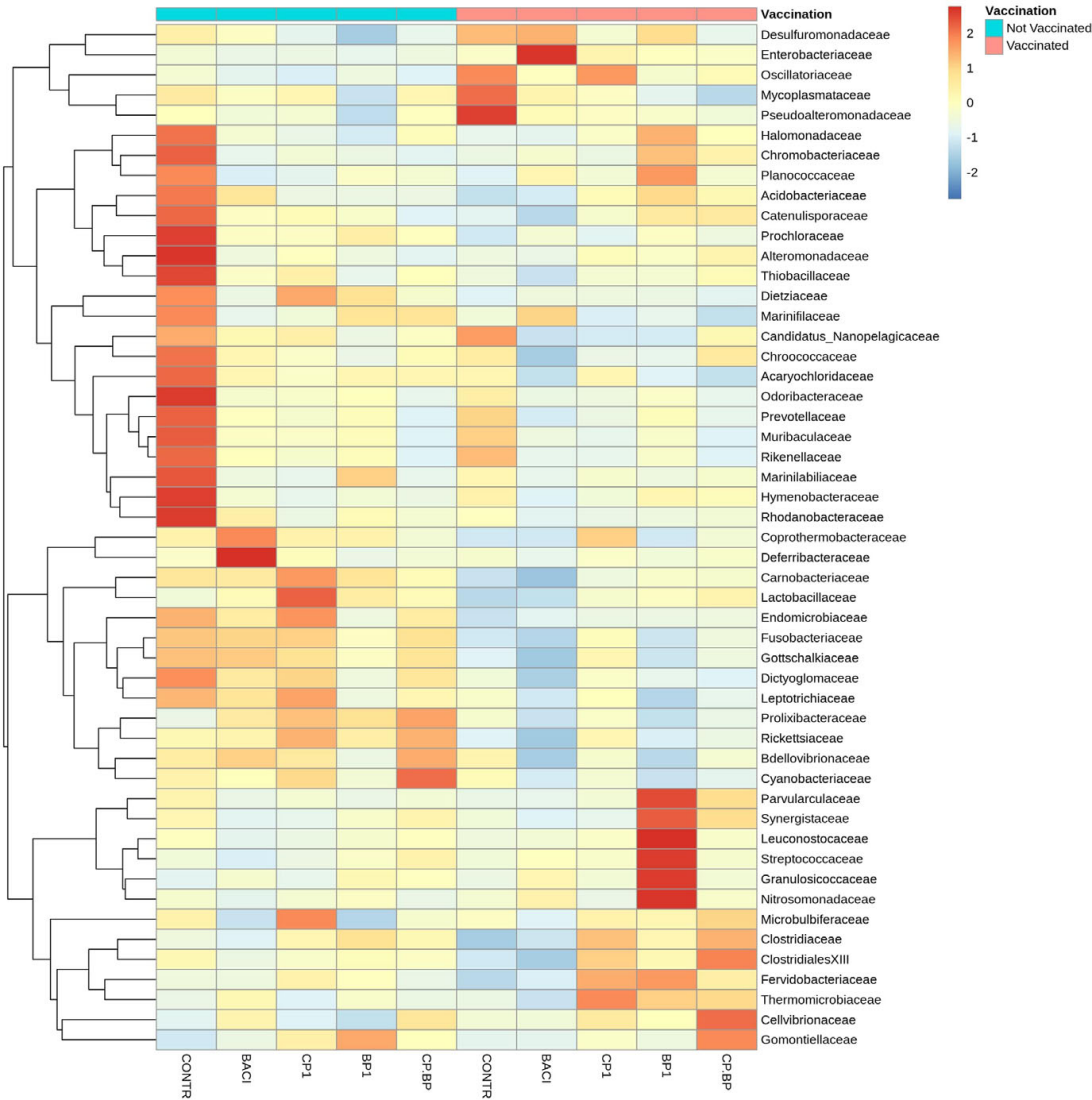
Cecal bacterial communities at the family level in broiler chicken fed bacitracin (55 ppm), 1% of each pomace of cranberry (CP1) and blueberry (BP1) or their combination (CP+BP). Non-vaccinated and vaccinated birds were labeled by blue and red colors, respectively at the top row.

As shown in the **Figure 5**, *Lactobacillaceae, Enterobacteriaceae, Clostridiaceae*, and *Streptococcaceae* were among the most abundant bacterial family in the ceca. These families were significantly (*P* < 0.05) abundant in CP1-fed birds regardless of vaccination as well as in vaccinated BACI- and BP1-fed birds (**Table 6**). For a given feed treatment, vaccination induced different effects on the relative abundances of *Acidobacteriaceae, Alteromonadaceae, Catenulisporaceae, Chromobacteriaceae, Deferribacteraceae, Leuconostocaceae*, *Marinifilaceae*, *Parvularculaceae*, *Planococcaceae, Thermomicrobiaceae*, and *Thiobacillaceae*. Ceca from non-vaccinated control birds receiving only a basal diet, showed an increased (*P* < 0.05) relative abundance of *Alteromonadaceae, Candidatus_Nanopelagicaceae*, and *Chroococcaceae*. The highest relative abundances of *ClostridialesXIII* (*P* = 0.0383) and *Gomontiellaceae* (*P* = 0.0242) were largely found in the CP+BP-fed vaccinated birds. Vaccination significantly reduced the relative abundance of *Lactobacillaceae*, which was increased (*P* = 0.01) in non-vaccinated CP1-fed birds (*P* = 0.04). However, the relative abundance of *Granulosicoccaceae* increased (*P* = 0.024) in vaccinated BP1-treated birds. Vaccination decreased (*P* < 0.05) the relative abundances of *Bdellovibrionaceae, Candidatus_Nanopelagicaceae, Dictyoglomaceae, Dietziaceae, Fusobacteriaceae, Gottschalkiaceae*, and *Lactobacillaceae* (**Table 6**).

**Table 6:** Microbial composition in the ceca digesta of 21-day-old broilers at family level significantly (*P* < 0.05) affected by studied dietary treatments.

Taxonomy (Family)	Vac	Treatments					SEM	*P* value		V X T
		Control	BACI	CP1	BP1	CP+BP		Vac	Treat	
*Acaryochloridaceae*	No	2.25	1.00	0.75	1.00	1.00	0.58	0.05	0.28	0.73
	Yes	1.00	0.00	1.00	0.25	0.00				
*Acidobacteriaceae*	No	202.75	155.25	108.25	107.50	106.75	30.40	0.46	0.93	0.04
	Yes	83.25	91.75	134.50	164.00	135.25				
*Alteromonadaceae*	No	261.75	83.75	111.00	82.00	66.75	37.65	0.42	0.15	0.03
	Yes	81.50	76.50	117.25	103.00	129.25				
*Bdellovibrionaceae*	No	55.50	65.00	57.75	37.00	71.25	12.45	0.01	0.25	0.49
	Yes	51.75	19.25	42.25	23.75	41.25				
*Candidatus_Nanopelagicaceae*	No	13.00	8.75	9.50	6.00	7.75	3.71	0.24	0.03	0.63
	Yes	13.75	4.00	4.50	4.50	8.75				
*Carnobacteriaceae*	No	158.25	154.75	191.25	160.75	141.25	33.00	0.02	0.80	0.85
	Yes	96.75	80.50	119.75	130.25	128.00				
*Catenulisporaceae*	No	19.25	10.00	11.00	9.50	6.75	3.13	0.34	0.47	0.05
	Yes	7.00	4.75	9.25	13.00	13.00				
*Cellvibrionaceae*	No	7.75	13.50	7.25	5.50	15.50	3.55	0.07	0.04	0.45
	Yes	10.25	9.75	15.00	12.25	23.00				
*Chromobacteriaceae*	No	348.25	201.25	219.00	208.50	197.75	38.41	0.88	0.38	0.05
	Yes	206.75	223.50	208.00	299.00	255.75				
*Chroococcaceae*	No	25.75	16.25	14.25	11.50	15.50	3.89	0.15	0.05	0.47
	Yes	17.75	6.50	11.50	11.25	18.25				
*Clostridiaceae*	No	237310.00	208052.00	298128.25	342950.75	293528.00	59969.75	0.90	0.02	0.45
	Yes	148517.50	181821.75	379548.50	297956.25	395040.75				
*ClostridialesXIII*	No	473.75	374.75	424.00	453.25	433.50	81.36	0.59	0.04	0.07
	Yes	308.75	245.00	581.25	473.50	690.00				
*Coprothermobacteraceae*	No	0.50	1.00	0.50	0.50	0.25	0.28	0.06	0.53	0.22
	Yes	0.00	0.00	0.75	0.00	0.25				
*Cyanobacteriaceae*	No	21.75	17.50	28.50	13.25	42.75	9.56	0.02	0.34	0.57
	Yes	19.00	5.00	14.00	3.00	8.75				
*Deferribacteraceae*	No	16.50	44.00	18.25	11.75	14.00	5.58	0.07	0.10	0.02
	Yes	15.00	11.00	16.00	13.75	15.50				
*Desulfuromonadaceae*	No	971.25	849.00	702.25	494.50	707.00	194.02	0.04	0.23	0.59
	Yes	1185.75	1214.75	808.50	1094.25	715.50				
*Dictyoglomaceae*	No	13.50	9.25	10.75	5.25	9.50	2.95	0.01	0.46	0.73
	Yes	5.50	1.50	6.50	4.50	3.75				
*Dietziaceae*	No	41.50	10.25	37.75	28.25	14.50	2.95	0.05	0.74	0.64
	Yes	6.75	12.25	11.00	10.50	7.75				
*Endomicrobiaceae*	No	9.25	7.00	10.00	4.50	7.00	2.22	0.01	0.80	0.63
	Yes	2.75	3.75	4.25	4.25	4.50				
*Enterobacteriaceae*	No	100131.50	25983.00	43137.75	7381.75	57096.00	220088.13	0.03	0.36	0.28
	Yes	166157.75	966716.75	286507.50	201041.25	162862.00				
*Fervidobacteriaceae*	No	51.00	49.25	80.00	68.00	46.50	27.77	0.29	0.08	0.44
	Yes	21.50	34.50	120.25	127.50	84.75				
*Fusobacteriaceae*	No	603.50	586.50	589.50	475.75	565.25	113.65	0.03	0.87	0.94
	Yes	373.75	344.00	494.50	368.00	435.00				
*Gomontiellaceae*	No	0.00	1.50	4.50	7.75	3.25	1.81	0.76	0.02	0.05
	Yes	1.00	1.00	2.75	1.75	8.75				
*Gottschalkiaceae*	No	112.50	109.50	101.25	80.25	100.25	18.42	0.00	0.61	0.59
	Yes	60.50	41.25	88.25	53.75	69.25				
*Granulosicoccaceae*	No	4.50	11.25	6.00	17.00	14.00	8.05	0.15	0.02	0.30
	Yes	7.00	17.50	9.75	46.50	10.00				
*Halomonadaceae*	No	493.25	300.00	271.50	239.75	330.00	70.37	0.91	0.64	0.07
	Yes	267.25	261.50	312.00	442.25	326.50				
*Hymenobacteraceae*	No	570.50	257.00	217.00	254.75	230.00	66.33	0.50	0.01	0.13
	Yes	328.75	192.75	248.00	315.50	300.00				
*Lactobacillaceae*	No	2358777.0	3138814.5	6050091.5	3645984.5	3153451.0	868133.08	0.01	0.04	0.31
	Yes	989362.00	1161741.8	2554653.3	2813498.8	3334950.5				
*Leptotrichiaceae*	No	216.75	193.75	226.75	142.00	172.75	45.01	0.06	0.54	0.99
	Yes	154.25	118.75	164.50	105.75	134.50				
*Leuconostocaceae*	No	476.75	141.00	230.50	369.25	497.75	236.18	0.04	0.01	0.01
	Yes	271.75	337.25	433.25	1834.50	412.50				
*Marinifilaceae*	No	2.75	0.50	0.75	1.75	1.75	0.54	0.03	0.23	0.02
	Yes	0.75	2.00	0.25	0.50	0.00				
*Microbulbiferaceae*	No	62.75	37.00	88.25	32.75	52.50	12.12	0.53	0.05	0.20
	Yes	55.50	43.00	63.50	61.75	74.00				
*Mycoplasmataceae*	No	675.75	591.50	630.00	439.75	634.50	82.90	0.82	0.01	0.17
	Yes	883.75	642.25	585.75	502.25	417.75				
*Nitrosomonadaceae*	No	15.50	10.50	14.75	17.00	11.75	5.28	0.03	0.02	0.06
	Yes	14.50	21.25	11.75	44.00	16.00				
*Odoribacteraceae*	No	414.75	79.00	82.50	107.25	22.50	73.42	0.18	0.02	0.53
	Yes	160.75	35.00	44.25	88.00	26.00				
*Oscillatoriaceae*	No	8.50	4.50	2.75	6.75	3.00	6.31	0.02	0.58	0.66
	Yes	28.00	11.25	26.50	9.25	12.75				
*Parvularculaceae*	No	12.50	3.25	6.50	3.00	5.75	5.38	0.08	0.11	0.02
	Yes	3.50	2.75	6.00	34.50	19.00				
*Planococcaceae*	No	526.00	168.50	198.75	273.25	253.50	90.51	0.76	0.30	0.04
	Yes	183.25	321.50	245.50	504.00	255.75				
*Prevotellaceae*	No	470.25	219.00	194.75	233.25	122.50	84.60	0.36	*0.04*	0.88
	Yes	335.50	106.25	154.75	232.50	146.50				
*Prochloraceae*	No	39.75	17.50	17.50	22.25	18.00	6.33	0.03	*0.59*	0.20
	Yes	9.00	15.75	11.25	17.50	13.75				
*Prolixibacteraceae*	No	3.75	8.25	10.75	9.25	12.00	2.94	0.01	*0.63*	0.50
	Yes	5.25	1.75	5.75	1.50	3.75				
*Pseudoalteromonadaceae*	No	19.75	16.75	17.50	11.00	19.25	4.31	0.05	*0.86*	0.41
	Yes	36.50	20.50	19.00	18.25	16.75				
*Rhodanobacteraceae*	No	177.25	101.50	65.75	90.25	74.50	21.53	0.03	*0.04*	0.23
	Yes	85.50	58.00	64.50	67.00	74.25				
*Rickettsiaceae*	No	51.75	53.75	75.00	56.75	74.50	14.98	0.01	*0.35*	0.96
	Yes	32.25	17.00	53.00	30.25	37.00				
*Rikenellaceae*	No	5461.25	1746.50	1252.00	1846.75	180.00	1365.12	0.42	*0.04*	0.99
	Yes	3927.25	562.00	573.50	1430.25	191.25				
*Thermomicrobiaceae*	No	4.75	7.75	3.75	6.25	5.00	2.36	0.05	0.34	0.04
	Yes	5.00	2.75	13.50	10.75	10.50				
*Thiobacillaceae*	No	15.75	5.00	7.25	2.75	5.75	2.23	0.02	0.04	0.04
	Yes	3.75	0.75	4.50	4.50	6.25				

Significant interactions were observed between dietary treatments and vaccination on the relative abundances of *M_capricolum* (*P* = 0.010) and *Agalactiae* (*P* = 0.047) from the *Mycoplasmataceae* and *Streptococcaceae* families, respectively. Furthermore, *Citrobacter, Enterobacter, Escherichia marmotae, Klebsiella aerogenes, Klebsiella michiganensis, Shigella flexneri, Ctenarytaina eucalypti*, and *Kosakonia cowanii* from the *Enterobacteriaceae* family also showered interactions between dietary treatments and vaccination (*P* < 0.05). This family was impacted by vaccination (*P* = 0.02) with the highest relative abundance of *E. coli* being observed in the vaccinated group particularly with the BACI fed birds compared to others. The highest abundance of *Lactobacillus crispatus* (*P* < 0.05), was found in CP1-terated birds from the non-vaccinated group. In addition to an overall increase of *Firmicutes*, blueberry pomace in feed increased the cecal abundance of *Streptococcaceae* in studied birds vaccinated against coccidiosis.

**Figure 6:**
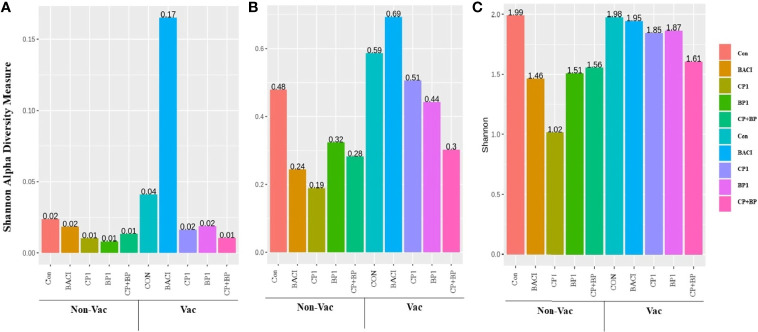
Shannon alpha diversity of 21-days old chicken ceca **(A)** at the domain level, **(B)** at the phylum level, and **(C)** at the family level. Shannon alpha diversity was significantly decreased in CP1-fed birds from the non-vaccinated group at the family level compared to controls (*P* = 0.047).

Alpha diversity measurements for different treatments in both non-vaccinated and vaccinated groups are presented in **Figure 6**. The higher abundances observed in all three taxonomic levels for BACI-treatment in the vaccinated group, were not statistically significant. At the family level, PERMANOVA test results using Bray-Curtis distances indicated a significant difference between dietary treatment. and interaction between treatments and vaccination were significant for Shannon Diversity Index. At the family level, Tukey Honest Significant Differences showed that the Shannon diversity Index (P = 0.047) decrease in CP1 fed birds compared to control from the non-vaccinated group.

## Discussion

Vaccination is a desirable approach that has been used during the last 50 years in the poultry industry to prevent some infectious diseases such as coccidiosis, Marek’s disease (27). In this study, a coccidiosis vaccine was administrated to broiler chicken fed berry pomaces to get insights of changes in gut health and immune response in a 30-day trial. Overall, no statistically significant effects of vaccination were observed in improving cumulative BW, ADG, or FE in birds when compared to non-vaccinated groups. However, during the growing phase from day 10 to 20, except for bacitracin fed birds, the coccidiosis vaccination the BW and the immune organs weight. The reduced performance parameters due to vaccination against coccidiosis was reported before (27). During the early stages of growth (day 1-21), live oocytes of the vaccine may replicate in the host, cause mild subclinical coccidiosis which is associated with a reduced absorptive area of the intestinal epithelium, malabsorption and inflammation (28). Thymus, spleen, liver and bursa of Fabricius are four important immune organs in poultry; an increase in the mass of these organs often indicates immune status of chickens (29). In the present study, birds receiving a vaccine against coccidiosis showed a reduced bodyweight along with the weight of all four organs mentioned above. However, no vaccination effect was noted on the ratio of organ weight/bodyweight. Body weight gain improvement can be impacted by immune stimulation because more nutrients will be repartitioned to synthesize antibodies and develop the immune organs, thereby decreasing the nutrients available for growth (30). Vaccination may affect the mucous membrane of epithelial cells by increasing the mucin production that may reduce the growth performance of birds in the first three weeks of age (31). An animal protein-rich diet was used in the present study and interactions of polyphenols with animal proteins in the feed may have affected the digestibility, availability, and functionality of amino acids (32). Hence, the accumulation of small quantities of polyphenols in body tissues could explain in part the poor synergistic effects of the combination of berry pomaces (CP+BP) in the feed. However, proteins may also act as good carriers to transfer polyphenols in the lower gastrointestinal tract and protect the host from oxidative reactions (32). The higher mortality rate observed in CP+BP treated birds needs further investigations. Accordingly, the complex nature of berry pomace bio-active compounds in addition to their content of condensed tannins (with some concerns in regard to fats and proteins absorption) necessitates an in-depth understanding of the chemical composition and mechanism of actions of the involved phytochemicals in relation to their effects at the farm-gates performance.

In general, lower incidences of coccidiosis due to *E. maxima* and *E. tenella* were observed in this work, indicating healthy and functional epithelium layers of intestines. Lower lesion scores have been also correlated with efficient nutrient utilization and absorption of the intestine (33). Good hygiene and better management practices could contribute to the reduction of *Eimeria* species (27). On the contrary, higher lesion scores of coccidiosis due to *E. acervulina* and NE due to *C. perfringens* were prevalent in the present study. Enteric diseases such as NE and coccidiosis are traditionally controlled by in-feed antimicrobials such as ionophores, and bacitracin (34). In the current study, bacitracin-treated birds showed mild lesion (score 1) with *E. acervulina* and *C. perfringens*, indicating subclinical coccidiosis possibly caused by the oocyst from the live coccidiosis vaccine. Despite the inclusion of animal protein in the diet, both CP1 and BP1 feed supplementation showed their potential in reducing *E. acervulina* and *C. perfringens* incidences similar to bacitracin as previously reported (20).

In the vaccinated group, oocysts shedding increased with the bird’s age. This increase of oocyst number could result in intestinal leak due to minor inflammations. In the non-vaccinated group, the significant reduction of oocyst counts in excreta was observed with the CP1 and (CP+BP) feed supplementation on both days 20 and 23. Berry polyphenol components such as flavonoids, proanthocyanidins (PAC), anthocyanins were reported to suppress the secretion of pro-inflammatory cytokines such as IL-4, a key regulator in humoral and adaptive immunity in human and rat models (35). These polyphenols could be a good source of antioxidants, which help in preventing incidences of coccidiosis (28). Incorporation of these potential “immunomodulatory” berry pomace extracts may enhance the coccidial specific humoral immunity and reduce fecal shedding. However, more research is required to understand the molecular mechanisms of berry pomace extracts in controlling coccidiosis.

Although the examination of different dietary approaches on chicken’s growth performance is available, their effects on blood metabolites in birds vaccinated against coccidiosis are limited. In the present study, vaccinated bird decreased serum calcium and phosphorus levels than the non-vaccinated birds. These two minerals are essential and have been reported to influence gut microbiota activity and immune system (36, 37). Changes of serum enzyme, lipid, and mineral levels have been reported in birds vaccinated against coccidiosis; however, varying results of these metabolites by different researchers indicates the necessity of considering other factors such as diet composition before setting any context for these changes (38). For example, Coweison (2020) did not observe any effect of vaccination on plasma mineral or GLU levels, while a lower AST concentration in vaccinated birds compared to non-vaccinated birds were reported (38). In the contrary, El-Maksoud et al. (39) reported vaccination effects on serum GLU, GLO, CHOL, HDLC, which were aligned with the current work. Further, the concentrations of serum enzymes ALT, and AST increased while levels GGT and other lipid molecules decreased with vaccination. The lack or reduction of different serum enzyme levels in the presence of various feed supplements is an indicator of healthy liver function (40). However, vaccination with live coccidial oocysts may induce immunity similar to coccidiosis with the disruption of the epithelial layers and may result in reduced nutrient digestibility and absorption (31). Overall, complex interactions between dietary protein concentration and coccidial vaccination makes these biochemical changes difficult to compare across studies (40). The high polyphenol contents of blueberry pomaces such as flavonols, anthocyanins, and antioxidant activities may improve hyperglycemia and dyslipidemia in animal models (41). Organic blueberry pomace has higher flavonols (6.17 vs. 3.08 mg quercetin eq/g), anthocyanins (16.08 vs 4.75 mg/g) contents and antioxidant activity (222 vs 144 µmol/Trolox eq. per g) than cranberry (17). Moreover, sterols and stilbenes contents of blueberry could be effective in lowering cholesterol and provide cardiovascular protection (42). It has been reported that blueberry extracts activated protein kinase through the activation of 50-adenosine monophosphate that contribute to glucose metabolism and insulin sensitivity (41). These studies could explain the observed reduction of glucose levels and lipid metabolites like TRIG and NEFA in the current experiments. Blueberry anthocyanins have been reported to exhibit a direct effect on the liver by reducing glucose production by 24–74% in H4IIE hepatocytes (41). Cranberry pomace contains relatively higher percentage values of the tannins than blueberry pomace (24.24 and 21.86 mg gallic acid eq/g, respectively) (17). Tannins are well-known for their antimicrobial and also anticoccidials activities; however, their combination with attenuated anticoccidial vaccines may reduce the effectiveness of vaccination by reducing nutrient absorption (31).

At present, accurate assessment of vaccine efficacy against *Eimeria* and the control of coccidiosis is lacking as no specific immunological assays are available to predict and measure the efficacy of a vaccine against coccidiosis (5). Administration of antibiotics may reduce the immune responses such as IgG antibody titers in humans, which can be recovered by supplementation of beneficial bacteria (43). In the present study, serum IgG (IgY), IgM, and IgA levels were determined under various feed treatments to inform on possible changes in bird’s humoral responses. Regardless of feed treatment, data showed a high immunoglobulin levels in vaccinated birds. However, serum antibody levels seem to be not enough to provide protection against *Eimeria* infection; cell-mediated responses to vaccination could be required to assess vaccine efficacy in broilers (5). Berry polysaccharides may act as immunomodulators of both innate and adaptive immunity, including cellular and humoral responses (31). Accordingly, dietary grape seed polyphenols, such as procyanidins, significantly increased IgG and IgM concentrations in weaned piglets (12). Feed supplementation with flavonoid-rich extracts such as oregano, thyme, and essential oils improved the serum IgY titer in broilers (44). Both cranberry and blueberry fractions contained a similar composition of oligomeric proanthocyanidins (PACs) and flavonols which could modulate the humoral immunity in broilers (42).

Chicken gut is inhabited by trillions of microbes, which benefits the host in many ways including modulation of the immune responses. However, limited information is available on the role of chicken gut microbiota and their responses to vaccination under different feeding regiments. In humans, differences in gut microbial compositions showed differences in responses to various vaccines (45). Germ-free or antibiotic-treated mice have shown impaired humoral responses to seasonal influenza vaccines (46). The administration of either the combination of *Lactobacillus* cocktail or fecal microbial transplant in antibiotic-treated layer chickens induced cell mediated immune responses such as the expression of IFN-γ (43). Vaccination enhanced the titers of immunoglobulins in the current study, while no significant differences were observed for any dietary treatment as stated above.

Chicken ceca comprise a diverse collection of microbial population which utilize carbohydrates over proteins as substrates for fermentation (10). Carbohydrates and fibers in blueberry and cranberry pomaces (17) can be fermented by the cecal bacteria to produce short chain fatty acids (SCFA) such as lactate, butyrate, while reducing ammonia production (10). The hydrolytic fragmentation of non-starch polysaccharides (NSP) could encourage the growth of beneficial bacteria families such as *Lachnospiraceae* and *Ruminococcaceae*. These two families were dominant in ceca allowing the utilization of recalcitrant polysaccharides to improve the overall energy production and absorption from the diet (47). In the present study, the effects of the berry pomace on *Firmicutes* was likely driven by an increase in *Lactobacillaceae* as fifty different species of *Lactobacillaceae* were observed among all treatments. The significant abundances of *Clostridiaceae* and *ClostridialesXIII* along with the higher incidences of *C. perfringens* could be reasons of poor performances of birds in CP+BP treatment from the vaccinated group.

*Lactobacillales* were the most dominant taxa (83%) of the entire cecal microbiota among treatments. This may have important implications for the improvement of broiler industry without the use antibiotic growth promoters. *Bdellovibrio bacteriovorus*, a highly motile *Proteobacteria* that prey on other Gram-negative bacteria, was found largely in the non-vac group particularly with the BACI and CP+BP treatment groups (**Table 6**). Because of its dual foraging strategy *B*. *bacteriovorus* was suggested as a living antibiotic in treating human infections (48).

## Conclusion

Feed supplementation with bacitracin and berry pomaces with or without a coccidiosis vaccination resulted in significant changes of growth performance and cecal microbiota profiles. Vaccination against coccidiosis is used to prevent incidences of *Eimeria* infections. However, this vaccination could negatively affect growth performance, particularly during the growing phase in broiler which could be addressed with cost efficient feed additives. Data from the present study indicated that coccidiosis vaccine perform better when used with bacitracin. Dietary blueberry pomace influenced several blood metabolites level. Interestingly, cranberry pomace in feed induced comparable effects as bacitracin on BW and studied enteric disease incidences in the absence of coccidiosis vaccination. Beside of its phenolic contents described above, berries are important sources of vitamin C which is known for its antioxidant and anti-inflammatory activities (42). The overall pleiotropic effects observed in the present study could be due to synergistic activities of various compounds found in the used pomaces. Further studies on the microbial metabolic functions with microbial populations and their correlation with growth performance parameters may provide insights on benefits related to the use of berry pomaces feed supplementation in broiler.

## Data Availability Statement

The illumina sequence read data of 40 cecum bacterial metagenomes from current study has been submitted to the Sequence Read Archive (SRA) database of the National Center for Biotechnology Information as FASTQ files under study accession number PRJNA666163.

## Ethics Statement

All experimental procedures performed in this study were approved (protocol #16-AV-314) by the Animal Care Committee of CRSAD, according to guidelines described by the Canadian Council on Animal Care (49).

## Author Contributions

MSD and QD conceived and designed the experiments. JS, MG, T-LH, CJ, XY, HY, QD, and MD performed the experiments and data analysis. MD, KR, and YK contributed reagents and materials. QD wrote the paper. MD, KR, JS, MG, KW, and MM reviewed and edited the manuscript. All authors contributed to the article and approved the submitted version.

## Funding

This work was financially supported by Agriculture and Agri-Food Canada through the Organic Science Cluster II program (#AIP CL-02 AGR-10383), the Canadian Federal Genomic Research and Development Initiative on Antimicrobial resistance (GRDI-AMR PSS #1858, J-001262) and OMFRA HQP program.

## Conflict of Interest

The authors declare that the research was conducted in the absence of any commercial or financial relationships that could be construed as a potential conflict of interest.

## References

[1] Gaucher M Quessy S Letellier A Arsenault J Boulianne M. Impact of a Drug-Free Program on Broiler Chicken Growth Performances, Gut Health, *Clostridium perfringens* and *Campylobacter jejuni* Occurrences at the Farm Level. Poultry Sci (2015) 94:1791–801. 10.3382/ps/pev142 26047674

[2] Agunos A Deckert A Léger D Gow S Carson C. Antimicrobials Used for the Therapy of Necrotic Enteritis and Coccidiosis in Broiler Chickens and Turkeys in Canada, Farm Surveillance Results (2013–2017). Avian Dis (2019) 63:433–45. 10.1637/11971-091718-Reg.1 31967426

[3] Ao X Kim IH. Effects of Grape Seed Extract on Performance, Immunity, Antioxidant Capacity, and Meat Quality in Pekin Ducks. Poultry Sci (2020) 99:2078-86. 10.1016/j.psj.2019.12.014 PMC7587615 32241493

[4] Lee J-W Kim D-H Kim Y-B Jeong S-B Oh S-T Cho S-Y. Dietary Encapsulated Essential Oils Improve Production Performance of Coccidiosis-Vaccine-Challenged Broiler Chickens. Animals (2020) 10:481. 10.3390/ani10030481 PMC7142951 32183035

[5] Soutter F Werling D Tomley FM Blake DP. Poultry Coccidiosis: Design and Interpretation of Vaccine Studies. Front Vet Sci (2020) 7:101. 10.3389/fvets.2020.00101 32175341 PMC7054285

[6] Kim WH Chaudhari AA Lillehoj HS. Involvement of T Cell Immunity in Avian Coccidiosis. Front Immunol (2019) 10:2732. 10.3389/fimmu.2019.02732 31824509 PMC6886378

[7] Williams RB. Intercurrent Coccidiosis and Necrotic Enteritis of Chickens: Rational, Integrated Disease Management by Maintenance of Gut Integrity. Avian Pathol (2005) 34:159–80. 10.1080/03079450500112195 16191699

[8] Adhikari P Kiess A Adhikari R Jha R. An Approach to Alternative Strategies to Control Avian Coccidiosis and Necrotic Enteritis. J Appl Poultry Res (2020) 29:515–34. 10.1016/j.japr.2019.11.005

[9] Boulton K Nolan MJ Wu Z Riggio V Matika O Harman K. Dissecting the Genomic Architecture of Resistance to *Eimeria Maxima* Parasitism in the Chicken. Front Genet (2018) 9:528. 10.3389/fgene.2018.00528 30534137 PMC6275401

[10] Yadav S Jha R. Strategies to Modulate the Intestinal Microbiota and Their Effects on Nutrient Utilization, Performance, and Health of Poultry. J Anim Sci Biotechnol (2019) 10:2. 10.1186/s40104-018-0310-9 30651986 PMC6332572

[11] Lee S Keirsey KI Kirkland R Grunewald ZI Fischer JG de La Serre CB. Blueberry Supplementation Influences the Gut Microbiota, Inflammation, and Insulin Resistance in High-Fat-Diet-Fed Rats. J Nutr (2018) 148(2):209–19. 10.1093/jn/nxx027 PMC6251676 29490092

[12] Lipiński K Mazur M Antoszkiewicz Z Purwin C. Polyphenols in Monogastric Nutrition - A Review. Ann Anim Sci (2017) 17:41–58. 10.1515/aoas-2016-0042

[13] Anhê FF Roy D Pilon G Dudonné S Matamoros S Varin TV. A Polyphenol-Rich Cranberry Extract Protects From Diet-Induced Obesity, Insulin Resistance and Intestinal Inflammation in Association With Increased *Akkermansia* Spp. Population in the Gut Microbiota of Mice. Gut (2015) 64:872–83. 10.1136/gutjnl-2014-307142 25080446

[14] Ghanei N Saghazadeh A Rezaei N. Gut Microbiome and Immunity. In: Mahmoudi M, Rezaei N. (eds) Nutrition and Immunity. Cham: Springer (2019). 10.1007/978-3-030-16073-9_10

[15] Cheng Y Wu T Chu X Tang S Cao W Liang F. Fermented Blueberry Pomace With Antioxidant Properties Improves Fecal Microbiota Community Structure and Short Chain Fatty Acids Production in an *In Vitro* Mode. LWT (2020) 125:109260. 10.1016/j.lwt.2020.109260

[16] Islam MR Hassan YI Das Q Lepp D Hernandez M Godfrey DV. Dietary Organic Cranberry Pomace Influences Multiple Blood Biochemical Parameters and Cecal Microbiota in Pasture-Raised Broiler Chickens. J Funct Foods (2020) 72:104053. 10.1016/j.jff.2020.104053

[17] Ross K Ehret D Godfrey D Fukumoto L Diarra M. Characterization of Pilot Scale Processed Canadian Organic Cranberry (Vaccinium Macrocarpon) and Blueberry (Vaccinium Angustifolium) Juice Pressing Residues and Phenolic-Enriched Extractives. Int J Fruit Sci (2017) 17:202–32. 10.1080/15538362.2017.1285264

[18] Canadian Council on Animal Care. CCAC Guidelines on the Care and Use of Farm Animals in Research, Teaching and Testing. Ottawa, ON: Canadian Council on Animal Care (2009). Available at: http://www.ccac.ca/en/standards/guidelines.

[19] Yang C Kennes YM Lepp D Yin X Wang Q Yu H. Effects of Encapsulated Cinnamaldehyde and Citral on the Performance and Cecal Microbiota of Broilers Vaccinated or Not Vaccinated Against Coccidiosis. Poultry Sci (2020) 99:936–48. 10.1016/j.psj.2019.10.036 PMC7587813 32029170

[20] Das Q Islam M Lepp D Tang J Yin X Mats L. Gut Microbiota, Blood Metabolites, and Spleen Immunity in Broiler Chickens Fed Berry Pomaces and Phenolic-Enriched Extractives. Front Vet Sci (2020) 7:150. 10.3389/fvets 33134328 PMC7188780

[21] Cobb Breeder Management Guide. (2016). Available at: https://www.cobb-vantress.com.

[22] Hodgson JN. Coccidiosis: Oocyst Counting Technique for Coccidiostat Evaluation. Exp Parasitol (1970) 28:99–102. 10.1016/0014-4894(70)90073-1 5459879

[23] Islam MR Oomah DB Diarra MS. Potential Immunomodulatory Effects of Non-Dialyzable Materials of Cranberry Extract in Poultry Production. Poultry Sci (2017) 96:341-50. 10.3382/ps/pew302 27587728

[24] Zaheer R Noyes N Polo RO Cook SR Marinier E Van Domselaar G. Impact of Sequencing Depth on the Characterization of the Microbiome and Resistome. Sci Rep (2018) 8:1–11. 10.1038/s41598-018-24280-8 29651035 PMC5897366

[25] Zaheer R Lakin SM Polo RO Cook SR Larney FJ Morley PS. Comparative Diversity of Microbiomes and Resistomes in Beef Feedlots, Downstream Environments and Urban Sewage Influent. BMC Microbiol (2019) 19:197. 10.1186/s12866-019-1548-x 31455230 PMC6712873

[26] SAS Institute Inc., Base SAS® 9.4 Procedures Guide. (2011), SAS Institute Inc. Cary, NC.

[27] Lee JT Eckert NH Ameiss KA Stevens SM Anderson PN Anderson SM. The Effect of Dietary Protein Level on Performance Characteristics of Coccidiosis Vaccinated and Nonvaccinated Broilers Following Mixed-Species Eimeria Challenge. Poultry Sci (2011) 90:1916–25. 10.3382/ps.2011-01362 21844255

[28] Quiroz-Castañeda RE Dantán-González E. Control of Avian Coccidiosis: Future and Present Natural Alternatives. BioMed Res Int (2015) 2015:430610. 10.1155/2015/430610 25785269 PMC4346696

[29] Guo M Hao G Wang B Li N Li R Wei L. Dietary Administration of Bacillus Subtilis Enhances Growth Performance, Immune Response and Disease Resistance in Cherry Valley Ducks. Front Microbiol (2016) 7:1975. 10.3389/fmicb.2016.01975 28008328 PMC5143344

[30] Liu Y-Y Wang Y Walsh TR Yi L-X Zhang R Spencer J. Emergence of Plasmid-Mediated Colistin Resistance Mechanism MCR-1 in Animals and Human Beings in China: A Microbiological and Molecular Biological Study. Lancet Infect Dis (2015) 16:161-8. 10.1016/S1473-3099(15)00424-7 26603172

[31] Arczewska-WŁOsek A ŚWiĄTkiewicz S. Nutrition as a Modulatory Factor of the Efficacy of Live Anticoccidial Vaccines in Broiler Chickens. World’s Poultry Sci J (2014) 70:81–92. 10.1017/S0043933914000075

[32] Jakobek L. Interactions of Polyphenols With Carbohydrates, Lipids and Proteins. Food Chem (2015) 175:556–67. 10.1016/j.foodchem.2014.12.013 25577120

[33] Calik A Omara II White MB Evans NP Karnezos TP Dalloul RA. Dietary non-Drug Feed Additive as an Alternative for Antibiotic Growth Promoters for Broilers During a Necrotic Enteritis Challenge. Microorganisms (2019) 7:257. 10.3390/microorganisms7080257 PMC6723652 31412542

[34] Lillehoj H Liu Y Calsamiglia S Fernandez-Miyakawa ME Chi F Cravens RL. Phytochemicals as Antibiotic Alternatives to Promote Growth and Enhance Host Health. Vet Res (2018) 49:1-18. 10.1186/s13567-018-0562-6 30060764 PMC6066919

[35] González-Gallego J García-Mediavilla MV Sánchez-Campos S Tuñó MJ. Fruit Polyphenols, Immunity and Inflammation. Br J Nutr (2010) 104(Suppl. 3):S15–27. 10.1017/S0007114510003910 20955647

[36] Rodriguez-Lecompte JC Yitbarek A Cuperus T Echeverry H van Dijk A. The Immunomodulatory Effect of Vitamin D in Chickens Is Dose-Dependent and Influenced by Calcium and Phosphorus Levels. Poult Sci (2016) 95:2547–56. 10.3382/ps/pew186 27252374

[37] Metzler-Zebeli BU Mann E Schmitz-Esser S Wagner M Ritzmann M Zebeli Q. Changing Dietary Calcium-Phosphorus Level and Cereal Source Selectively Alters Abundance of Bacteria and Metabolites in the Upper Gastrointestinal Tracts of Weaned Pigs. Appl Environ Microbiol (2013) 79:7264. 10.1128/AEM.02691-13 24038702 PMC3837733

[38] Cowieson AJ Livingston ML Nogal B Hoang V Wang YT Crespo R. Effect of Coccidial Challenge and Vaccination on the Performance, Veterinary Postmortem Scores, and Blood Biochemistry of Broiler Chickens. Poultry Sci (2020) 99:3831–40. 10.1016/j.psj.2020.05.018 PMC7598015 32731969

[39] El-Maksoud A Afaf H Abdel-Magid D El-Badry M. Biochemical Effect of Coccidia Infestation in Laying Hen. Benha Vet Med J (2014) 26:127–33.

[40] Arczewska-Włosek A Świątkiewicz S Ognik K Józefiak D. Effect of Dietary Crude Protein Level and Supplemental Herbal Extract Blend on Selected Blood Variables in Broiler Chickens Vaccinated Against Coccidiosis. Animals (2018) 8:208. 10.3390/ani8110208 PMC6262617 30445686

[41] Shi M Loftus H McAinch AJ Su XQ. Blueberry as a Source of Bioactive Compounds for the Treatment of Obesity, Type 2 Diabetes and Chronic Inflammation. J Funct Foods (2017) 30:16–29. 10.1016/j.jff.2016.12.036

[42] Das Q Islam MR Marcone MF Warriner K Diarra MS. Potential of Berry Extracts to Control Foodborne Pathogens. Food Control (2017) 73:650–62. 10.1016/j.foodcont.2016.09.019

[43] Yitbarek A Astill J Hodgins DC Parkinson J Nagy É Sharif S. Commensal Gut Microbiota can Modulate Adaptive Immune Responses in Chickens Vaccinated With Whole Inactivated Avian Influenza Virus Subtype H9n2. Vaccine (2019) 37:6640–7. 10.1016/j.vaccine.2019.09.046 31542262

[44] Huang C Lee T. Immunomodulatory Effects of Phytogenics in Chickens and Pigs—A Review. Asian-Australas J Anim Sci (2018) 31:617. 10.5713/ajas.17.0657 29268586 PMC5930271

[45] Hagan T Cortese M Rouphael N Boudreau C Linde C Maddur MS. Antibiotics-driven Gut Microbiome Perturbation Alters Immunity to Vaccines in Humans. Cell (2019) 178:1313–28.e13. 10.1016/j.cell.2019.08.010 31491384 PMC6750738

[46] Ciabattini A Olivieri R Lazzeri E Medaglini D. Role of the Microbiota in the Modulation of Vaccine Immune Responses. Front Microbiol (2019) 10:1305. 10.3389/fmicb.2019.01305 31333592 PMC6616116

[47] Apajalahti J Vienola K. Interaction Between Chicken Intestinal Microbiota and Protein Digestion. Anim Feed Sci Technol (2016) 221:323–30. 10.1016/j.anifeedsci.2016.05.004

[48] Iebba V Totino V Santangelo F Gagliardi A Ciotoli L Virga A. Bdellovibrio Bacteriovorus Directly Attacks Pseudomonas Aeruginosa and Staphylococcus Aureus Cystic Fibrosis Isolates. Front Microbiol (2014) 5:280. 10.3389/fmicb.2014.00280 24926292 PMC4046265

[49] CCAC. Canadian Council on Animal Care Guidelines on: The Care and Use of Farm Animals in Research, Teaching and Testing. Ottawa, ON: Canadian Council on Animal Care (CCAC) (2009) Available online at: http://www.ccac.ca/Documents/Standards/Guidelines/Farm_Animals.pdf

